# Gearbox fault diagnosis method based on lightweight channel attention mechanism and transfer learning

**DOI:** 10.1038/s41598-023-50826-6

**Published:** 2024-01-07

**Authors:** Xuemin Cheng, Shuihai Dou, Yanping Du, Zhaohua Wang

**Affiliations:** Department of Mechanical and Electrical Engineering, Beijing Institute of Graphic Communication, Beijing, 102600 China

**Keywords:** Mechanical engineering, Computational science, Computer science, Information technology

## Abstract

In practical engineering, the working conditions of gearbox are complex and variable. In varying working conditions, the performance of intelligent fault diagnosis model is degraded because of limited valid samples and large data distribution differences of gearbox signals. Based on these issues, this research proposes a gearbox fault diagnosis method integrated with lightweight channel attention mechanism, and further realizes the cross-component transfer learning. First, time–frequency distribution of original signals is obtained by wavelet transform. It could intuitively reflect local characteristics of signals. Secondly, based on a local cross-channel interaction strategy, a lightweight efficient channel attention mechanism (LECA) is designed. The kernel size of 1D convolution is affected by channel number and coefficients. Multi-scale feature input is used to retain more detailed features of different dimensions. A lightweight convolutional neural network is constructed. Finally, a transfer learning method is applied to freeze lower structures of the network and fine-tune higher structures of the model using small samples. Through experimental verification, the proposed model could effectively utilize samples. The application of transfer learning could realize accurate and fast fault classification of small samples, and achieve good gearbox fault diagnosis effect under varying working conditions and cross-component conditions.

## Introduction

At present, mechanical equipment is widely used in industrial production and intelligent manufacturing. However, the operation environment of mechanical equipment is complex in practical applications. The long-term running could lead to aging and damage of components in mechanical equipment^1^. If critical components fail, the operation of equipment will be affected, resulting in huge losses.

Gearbox is a key transmission component in mechanical equipment. It is usually in a non-stationary and variable load operating environment. In early stages, weak fault of mechanical components is often ignored due to the interference of environmental and other noise. If allowed to progress, it could interfere with the normal operation of equipment and even lead to casualties and other accidents^2^. Therefore, the research of gearbox fault diagnosis can reduce the occurrence of major accidents. The research is of great meaning for enhancing the reliability and security of equipment operation.

The key points of gearbox fault diagnosis methods are signal feature extraction and fault pattern recognition. The main methods for feature extraction embrace Short Time Fourier Transform (STFT)^3^, Variational Mode Decomposition (VMD)^4^ and Wavelet Transform (WT)^5^, etc. Traditional pattern recognition algorithms mainly include Support Vector Machine (SVM)^6^, Sparse Representation Classification (SRC)^7^, Artificial Neural Network (ANN)^8^, etc.

With fault diagnosis gradually stepping into the big data era, the collected signals not only have a large amount, but also have complex and diverse types. Traditional identification methods are difficult to meet the demand in big data era. Intelligent identification of gearbox faults is a necessary research^9,10^. In recent years, deep learning^11^ is one of the fastest-growing domains in machine learning. It has found an increasingly wide utilization in fault diagnosis. In deep learning methods, convolutional neural network (CNN) utilizes the idea of weight sharing and local sense to decrease complexity and computational cost of network^12,13^. It has significant advantages 2D image classification. CNN is universally adopted in mechanical equipment fault diagnosis field due to the superior classification performance. For example, Ye et al.^14^ proposed a new intelligent rolling bearing fault diagnosis method based on variational mode extraction (VME) and improved 1D-CNN, which had strong feature learning ability. Long et al.^15^ used pixel filling method to convert signals into images and input these images into a 2D-CNN network to achieve high-precision fault classification. Yan et al.^16^ proposed a deep order-wavelet convolutional variational autoencoder (DOWCVAE) network to identify bearing faults under fluctuating speed conditions. The research could improve feature learning ability of a plain convolutional variational autoencoder. Zhang et al.^17^ designed a multi-branch residual convolutional neural network that achieved high-precision gearbox fault diagnosis.

Although CNN has strong feature extraction capabilities, the key information could be weaken when applying max or average pooling directly merges features in a model. The attention mechanism is a good solution to this problem^18,19^. Zhao et al.^20^ embedded an improved channel and spatial attention module in residual structure and focused attention on effective information of feature maps. Li et al.^21^ combined Dual-stage Attention-based Recurrent Neural Network (DA-RNN) and Convolutional Block Attention Module (CBAM) to obtain a bearing fault diagnosis model, which achieved good diagnosis results under unbalanced data condition. Liu et al.^22^ constructed a stacked residual multi-attention network (SRMANet) to take critical feature components of gearbox vibration signals. Zhao et al.^23^ presented a novel rotor system fault diagnosis model based on parallel convolutional neural network architecture with attention mechanism (AMPCNN), which had good performance for load adaptability and noise immunity. Ding et al.^24^ designed a feature-guided attention mechanism and embedded it into the residual network to enhance its generalization ability. Li et al.^25^ integrated the convolutional neural network (CNN) with attention mechanisms to strengthen the representational power of fault samples.

To sum up, the deep learning algorithm could adaptively extract fault features and has strong fault classification performance. However, large numbers of samples are usually required for fault diagnosis using deep learning algorithm. The monitored gearbox signals are mostly normal operation data. The fault samples are few. If samples are limited, the recognition precision and generalization capability of neural networks are weak. Transfer learning method can better solve such problems^26^.

Zheng et al.^27^ introduced open source bearing samples as source domain data and treated a target domain bearing dataset as small samples. Transfer learning model was refined by a new optimal fusion way. Dong et al.^28^ proposed a small sample intelligent bearing diagnosis method based on dynamic model and transfer learning, aiming at the difficulty of obtaining fault data in practical engineering. Yu et al.^29^ proposed a feature fusion CNN based on transfer learning. The network is of strong robustness and high accuracy verified by bearing fault diagnosis experiment. He et al.^30^ combined deep transfer learning method and improved residual shrinkage network to achieve cross-condition quantitative diagnosis of bearing faults. Li et al.^31^ proposed a planetary gears fault diagnosis approach based on intrinsic feature extraction and deep transfer learning. Zhong et al.^32^ proposed a novel fault diagnosis method based on incorporating data augmentation and fine-tuning transfer learning, which combined the synthetized samples and original data to train the deep network.

In summary, a transfer learning model only requires limited samples to train a network suitable for the current task. It solves the problem of lacking numerous labeled samples. However, the above researches only consider a fault diagnosis model on one type of component and did not consider the performance of models on different components. There are complex distribution differences in the fault signals generated by different components. Moreover, the effective gearbox fault samples are limited under variable operating conditions. The above problems lead to poor diagnostic performance and weak generalization ability of models.

Therefore, this research proposes a gearbox fault diagnosis method based on lightweight channel attention mechanism and transfer learning. The method could solve the above problems and realize accurate classification of limited gearbox samples under varying working conditions and cross-component conditions. The main contributions of this paper are as follows:

(1)A new model based on EfficientNetV2 network is proposed. It uses channel attention mechanism to optimize the negative impact of dimension reduction through appropriate cross channels. Multi-scale feature input is used to retain more detailed features of different dimensions. Based on a local cross-channel interaction strategy without dimensionality reduction, the size of cross-channel affected by channel number and coefficients is adjusted, which make the attention mechanism lightweight.

(2)A transfer learning strategy is applied to extract features from limited samples. The strategy achieves high-precision fault diagnosis for small samples of untrained working conditions and components. It expands the application range of transfer learning. Through experimental verification, the proposed model has strong generalization ability. It could fit the fault distribution difference in different working conditions and components. Simultaneously, it still has good fault diagnosis performance under limited samples.

The rest of this paper is organized as follow. In Section “Method”, the paper introduces wavelet transform method, lightweight network, channel attention mechanism and transfer learning in detail. In Section “Constructing gearbox fault diagnosis model”, a fault diagnosis model based on transfer learning and LECA module is designed, and detailed flow of fault diagnosis is shown. In Section “Experimental verification”, the comprehensive performance of the model is demonstrated taking the gearbox fault dataset published by Southeast University. Conclusions are presented in Section “Conclusion”.

## Method

### Wavelet transform

The signals could be directly displayed the mapping relationship in time-domain and frequency-domain using time–frequency analysis method. It could intuitively reflect the local characteristics of the signals. As one of the representative methods of time–frequency analysis, wavelet transform describes the time–frequency characteristics of raw signals by translating and stretching wavelet basis functions. It could flexibly change the window length according to the frequency amplitude and provide good resolution results for non-periodic signal. Wavelet transform can be expressed as the inner product of signal $x(t)$ and wavelet basis function ${\psi }_{a,b}(t)$. The expression is shown in Eq. (1):

1 $$WT(a,b)=\frac{1}{\sqrt{a}}{\int }_{-\infty }^{\infty }x(t)\psi \left(\frac{t-b}{a}\right)dt$$

2 $${\psi }_{a,b}(t)=\frac{1}{\sqrt{a}}\psi \left(\frac{t-b}{a}\right)$$

where $t$ is time variable, $b$ is a translation factor, $a$ is a stretching factor used to control the stretching size of the wavelet basis function.

### Lightweight convolutional neural networks

At present, gearbox fault diagnosis models can achieve high accuracy, but most of the models have complex structures and occupy a lot of computing resources. Therefore, the lightweight and high-precision model, EfficientNetV2, is selected as the basic network to reduce the computation cost.

**Figure 1:**
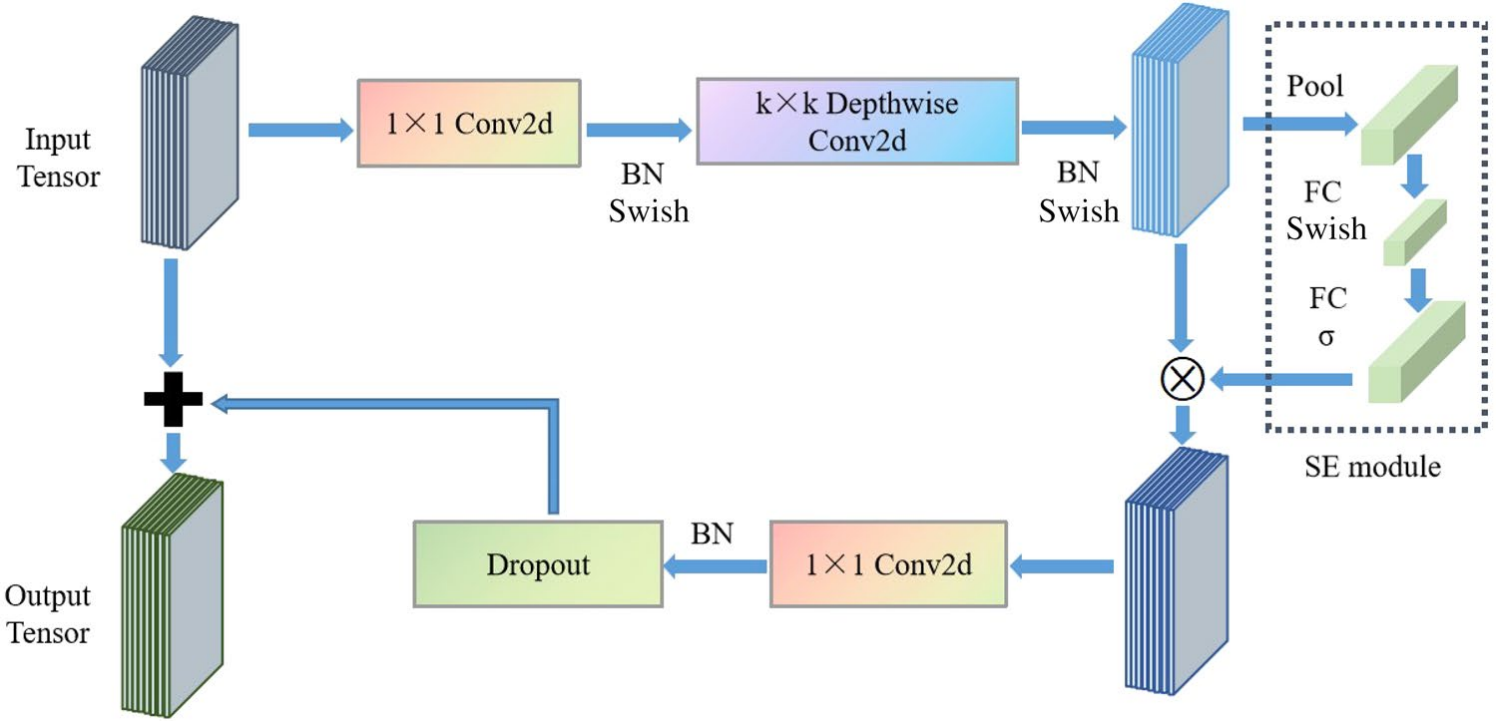
Architectural details of MBConv module.

**Figure 2:**
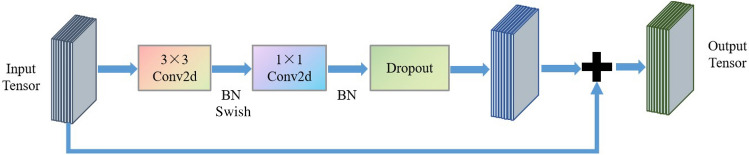
Architectural details of Fused-MBConv module.

EfficientNetV2 is a new lightweight CNN combining neural network search technology^33^. Its core structure is MBConv and Fused-MBConv module, as shown in Fig. 1 and 2. MBConv module is composed of depthwise separable convolution (DSC) and SE modules. MBConv module firstly raises the dimension, then calculates using DSC, and finally reduces dimension by convolution layer. Fused-MBConv replaces DSC with standard convolution to improve operating speed. Fused-MBConv module is used in lower structure of the network. MBConv is applied in the higher structure. It could reduce the network parameters amount and improve computational speed.

### Channel attention mechanism

Nowadays, attention mechanism has been extensively used in deep neural network due to its characteristics of sharing weights and strengthening effective information. To improve the ability to extract effective information from gearbox signals, this paper introduces a channel attention mechanism to optimize the performance of gearbox fault diagnosis model.

**Figure 3:**
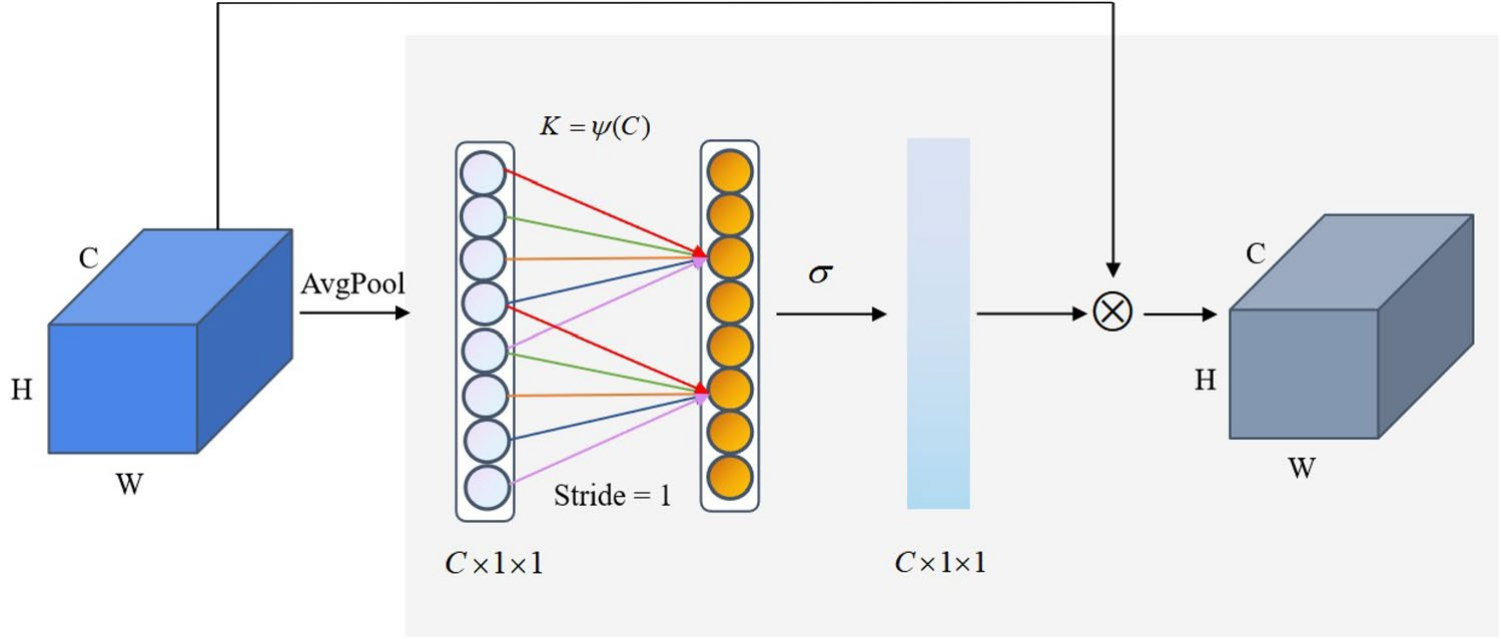
Architectural details of ECA module.

In SE modules, dimension reduction leads to a decline in model learning ability. Wang et al.^34^ proposed an efficient channel attention (ECA) mechanism for the above issue. The architectural details of ECA module is shown in Fig. 3. It adopts a local cross-channel interaction strategy without dimension reduction. The module obtains more accurate attention information using a 1D convolutional layer to aggregate cross-channel information. First, the aggregation features of each channel are obtained by global average pooling. Second, the kernel size K is adaptively calculated using the channel number C. Finally, the weight of each channel is calculated adopting 1D convolution and a sigmoid function.

### Transfer learning

Transfer learning is a machine learning method. It reapplies features learned from a task to a target task. In transfer learning, the domain to be learned is usually defined as source domain ${D}_{s}=\{{x}_{s},P({x}_{s})\}$, its learning task ${T}_{s}=\{{y}_{s},{f}_{s}(\cdot )\}$. The domain to be solved is called target domain ${D}_{t}=\{{x}_{t},Q({x}_{t})\}$, and its learning task ${T}_{t}=\{{y}_{t},{f}_{t}(\cdot )\}({D}_{s}\ne {D}_{t}\,\mathrm{ or }\,{T}_{s}\ne {T}_{t})$. Transfer learning is to acquire knowledge in ${D}_{s}$ and ${T}_{s}$ to help the learning of ${f}_{t}(\cdot )$^26^. The fault diagnosis effect of a deep learning model is closely related to whether the training samples are sufficient. Only based on many training samples can a high-precision deep learning model be obtained. The transfer learning method could realize the knowledge transfer. The knowledge learned from source domain with sufficient data is applied to target domain with few samples.

## Constructing gearbox fault diagnosis model

To preserve more detailed features in samples, this paper uses average pooling and max pooling to extract two different scales information of original signals. Average pooling reflects the global information of feature maps and provides feedback for each point on feature maps. Max pooling captures the local features of signals and presents the overall trend of signal change^35^.

**Figure 4:**
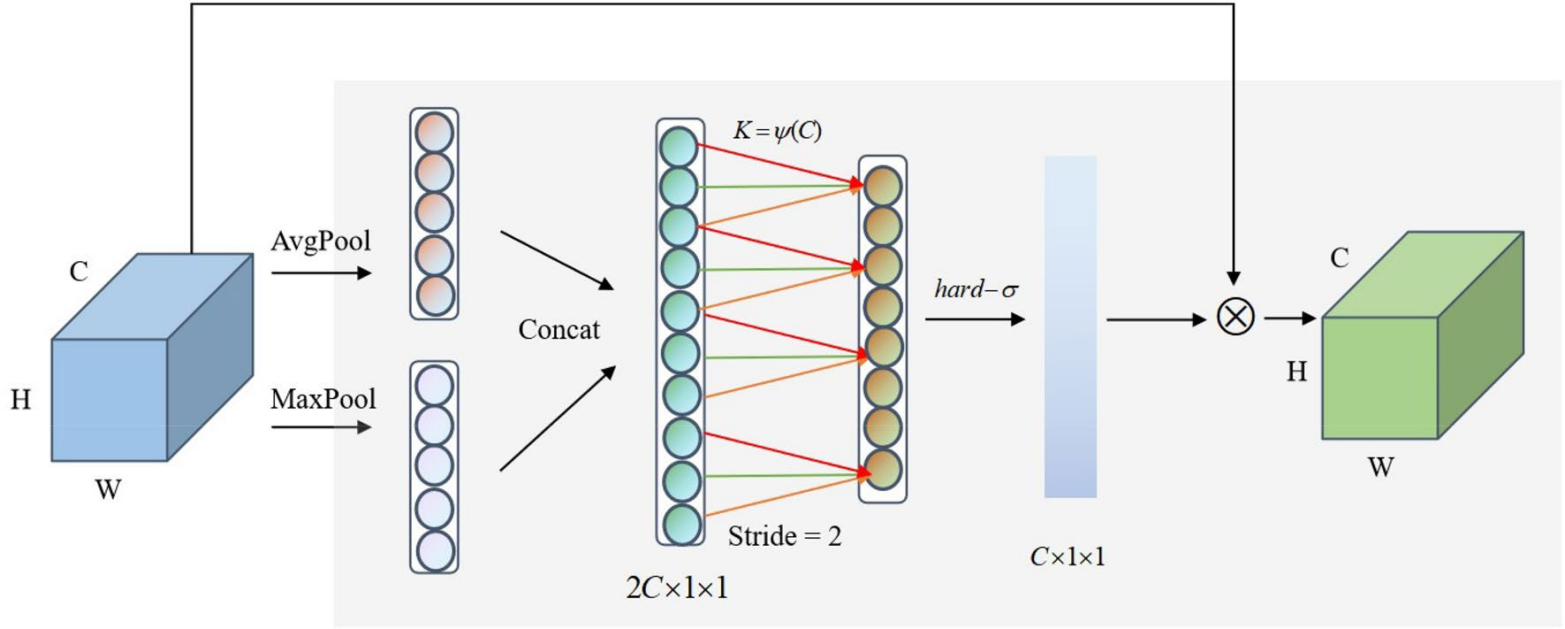
Architectural details of LECA module.

In this paper, the multi-scale features input is applied to take the global and local features of samples. A lightweight channel attention mechanism (LECA) is designed. The LECA consists of 1D convolutional layer, BN layer, max pooling, average pooling, and hard-sigmoid activation function. The LECA captures the dependency between channels by aggregating global and local features. It could adaptively calculate the kernel size according to channels number and coefficients. By reducing the most suitable even number related to channel coefficients, the attention mechanism is more lightweight. The stride is set to 2. The features learned by different convolution kernels are scored adaptively. The LECA architectural details is shown in Fig. 4. The expression for the channel attention mechanism is described as follows.

H and W represent the height and width of the input feature maps. C represents the number of channels. The expressions of average pooling and max pooling are shown in Eq. (3) and (4):

3 $${z}_{1}=\frac{1}{H\times W}{\sum }_{i=1}^{h}{\sum }_{j=1}^{w}f(i,j)$$

4 $${z}_{2}=\mathit{max}f(i,j)$$

where ${z}_{1}$ and ${z}_{2}$ are the outputs of global average pooling and global max pooling. $f$ are a set of 2D feature maps.

5 $$w=\sigma (C1D_k (y))$$

where $C1D_{k}$ represents 1D convolution operation with kernel size k, $\sigma$ is a hard-sigmoid activation function, and $W$ is the weight parameter.

6 $${\widetilde{X}}_{c}=w\otimes f(i,j)$$

where ${\widetilde{X}}_{c}$ is the optimized feature matrix. Its height, width and channel number are the same as the size of the input feature matrix. After adjusting the channel attention module, the important feature information will be enhanced.

In Fig. 4, K represents the coverage of local cross-channel interaction and also represents kernel size for 1 × 1 convolutional layer. To lighten the attention module, subtract the most suitable even number based on original ECA. The even number is affected by coefficients γ and b. There is a mapping relationship between K and channel C as follows:

7 $$K=\varphi (C)={\left|\frac{{\mathit{log}}_{2}(C)+b}{\gamma }\left.-{|\frac{\gamma }{b}|}_{even}\right|\right.}_{odd}$$

where ${\left|\frac{{\mathit{log}}_{2}(C)+b}{\gamma }\left.-{|\frac{\gamma }{b}|}_{even}\right|\right.}_{odd}$ represents the nearest odd number, the coefficients $\gamma$ and $b$ are set to 2 and 1, respectively.

The network proposed adopts MBConv and Fused-MBConv module in EfficientNetV2 network, replacing all SE modules with LECA modules. Since depthwise convolution is slow operation speed in shallow networks, the Fused-MBConv module is applied in the lower structures. The 3*3 convolution is selected to quickly extract signal features to match the size of time–frequency map. To overcome the gradient vanishing problem, the smooth SiLU function is selected as activation function. In the deep structure, the MBConv module is used. To reduce the negative impact of dimension reduction in SE, LECA module is integrated to evaluate the importance of different channel features, highlighting important features and inhibiting invalid features. The multi-scale feature input is used to save more detailed features in signals. Thus, the feature extraction capability and robustness of the model are enhanced. The detailed structure and parameter of model are shown in Table 1.

**Table 1:** Structure and parameter of LECA-EfficientNetV2 network.

Input	Operator	Exp size	Out	LECA	Activation Function	Stride	Padding
224^2^ × 3	Conv2d, 3 × 3	–	24	–	Swish	2	1
112^2^ × 24	Fused-MBConv, 3 × 3	24	24	–	Swish	1	1
112^2^ × 24	Fused-MBConv, 3 × 3	24	24	–	Swish	1	1
112^2^ × 24	Fused-MBConv, 3 × 3	96	48	–	Swish	2	1
56^2^ × 48	Fused-MBConv, 3 × 3	192	48	–	Swish	1	1
56^2^ × 48	Fused-MBConv, 3 × 3	192	64	–	Swish	2	1
28^2^ × 64	Fused-MBConv, 3 × 3	256	64	–	Swish	1	1
28^2^ × 64	Fused-MBConv, 3 × 3	256	64	–	Swish	1	1
28^2^ × 64	MBConv, 3 × 3	256	128	√	Swish	2	1
14^2^ × 128	MBConv, 3 × 3	512	128	√	Swish	1	1
14^2^ × 128	MBConv, 3 × 3	512	128	√	Swish	1	1
14^2^ × 128	MBConv, 3 × 3	512	128	√	Swish	1	1
14^2^ × 128	Conv2d, 1 × 1	–	1280	–	Swish	1	1
14^2^ × 1280	Pool, 14 × 14	–	1280	–	–	–	–
1^2^ × 1280	Fully-connected layer	–	5	–	–	–	–

**Figure 5:**
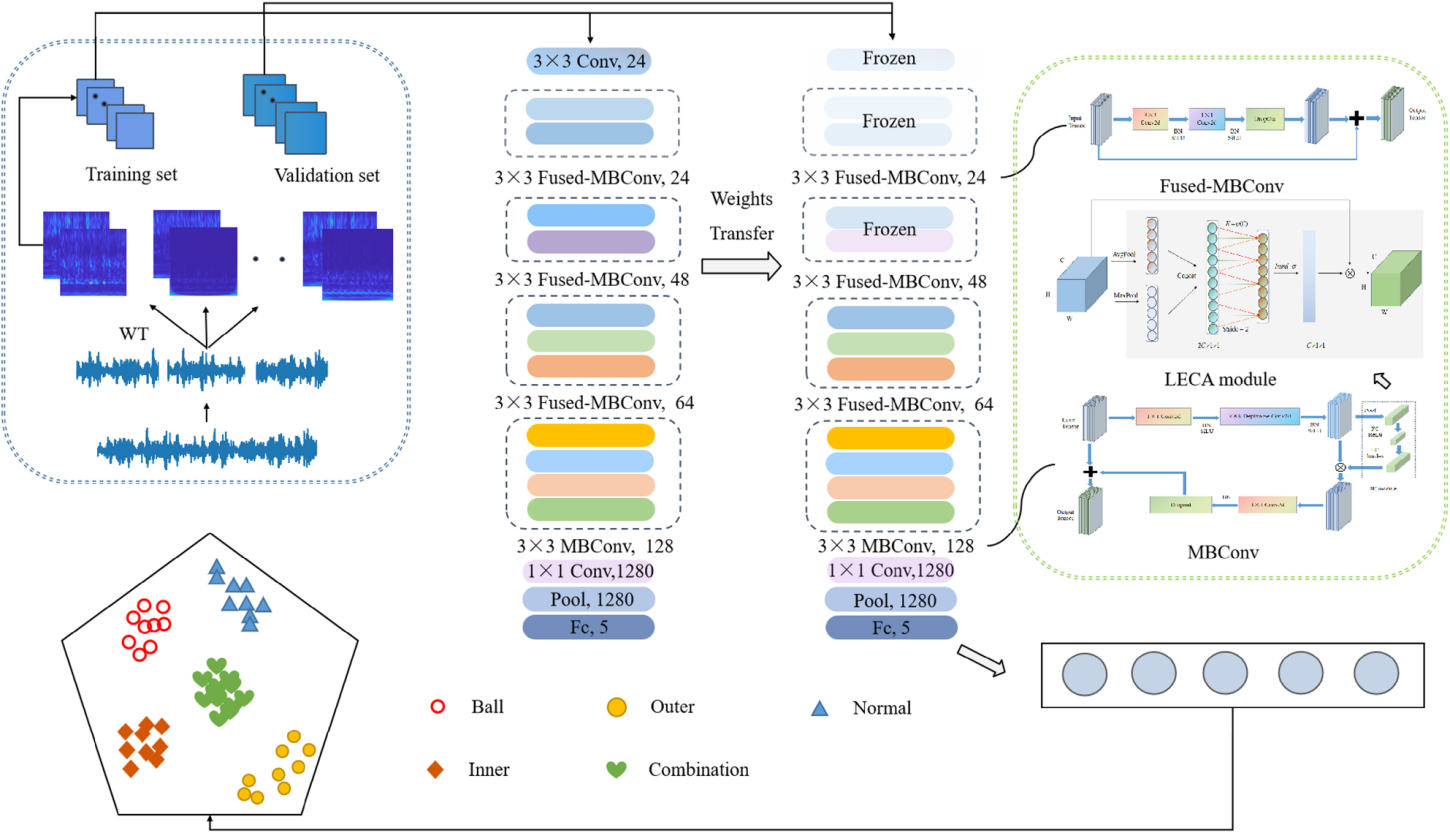
Fault diagnosis flow of the LECA-EfficientNetV2 transfer learning model.

The transfer learning is introduced into the research. In the process of transfer learning, the transfer effect of data from similar fields is better than that of two domains with significant differences. Under different working conditions, the samples of gearbox components have certain similarities. Therefore, the transferred parameters can be used as a powerful set of features to reduce the complexity and training time of the network. The fault diagnosis flow of the proposed method is shown in Fig. 5. The detailed implementation steps are as follows:

1. Sample pretreatment. Wavelet transform is performed on original signals to obtain RGB images (224 × 224 × 3). All the obtained samples are divided into training sets and validation sets, which are respectively used for training and evaluating the final effect of the model. One working condition is used as source domain samples. The other working conditions are used as target domain samples. The gearbox datasets are constructed under different working conditions.
2. Model training and transfer. The convolutional neural network is constructed for training. The model weight, learning rate and other parameters are determined according to training results. The LECA module is used to extract the key features of the fault signals. Freeze the lower structure of the network, including the first convolutional layer and four Fused-MBConv modules. Fine-tune the higher structure of the network with the small samples in different working conditions. The sample distribution difference caused by different working conditions is reduced.
3. Model application. Input validation samples from the target domain into the trained model. The Softmax classification layer is used to output the results to complete the gearbox fault diagnosis for varying working conditions. Furthermore, the cross-component fault diagnosis is further realized according to the above process.

## Experimental verification

### Data introduction

The gearbox dataset from Southeast University is used as fault diagnosis experimental data in this paper, which was obtained on drivetrain dynamic simulator (DDS)^26^. This platform is composed of a programmable controller, a variable speed drive motor, a two-stage planetary gearbox and a two-stage parallel shaft spur gearbox, etc. This dataset includes two sub datasets: bearing and gearbox datasets. Each sub dataset has one health state and four fault states. The detailed fault types are shown in Table 2.

**Table 2:** Bearing and gear failure types description and Dataset partitioning.

Subject	Type	Description	Source domain		Target domain		Working conditions
			training	validation	training	validation	
Bearing	Normal	Health state	640	160	50	150	20 Hz-0 V, 30 Hz-2 V
	Ball	Ball crack	640	160	50	150	20 Hz-0 V, 30 Hz-2 V
	Inner	Inner ring crack	640	160	50	150	20 Hz-0 V, 30 Hz-2 V
	Outer	Outer ring crack	640	160	50	150	20 Hz-0 V, 30 Hz-2 V
	Combination	Inner and outer ring crack	640	160	50	150	20 Hz-0 V, 30 Hz-2 V
Gear	Normal	Health state	640	160	50	150	20 Hz-0 V, 30 Hz-2 V
	Chipped	Teeth crack	640	160	50	150	20 Hz-0 V, 30 Hz-2 V
	Miss	Missing teeth	640	160	50	150	20 Hz-0 V, 30 Hz-2 V
	Root	Root crack	640	160	50	150	20 Hz-0 V, 30 Hz-2 V
	Surface	Surface wear	640	160	50	150	20 Hz-0 V, 30 Hz-2 V

The sample size of source domain is 4000, which is used to train the model. Each fault type consists of 800 samples, divided into a training set and a validation set in a 4:1 ratio. The sample size of target domain is 1000, which is used to verify the generalization and transfer effects for small samples. Each fault type consists of 200 samples, divided into a training set and a validation set in a 1:4 ratio. The detailed partitioning is shown in Table 2.

**Figure 6:**
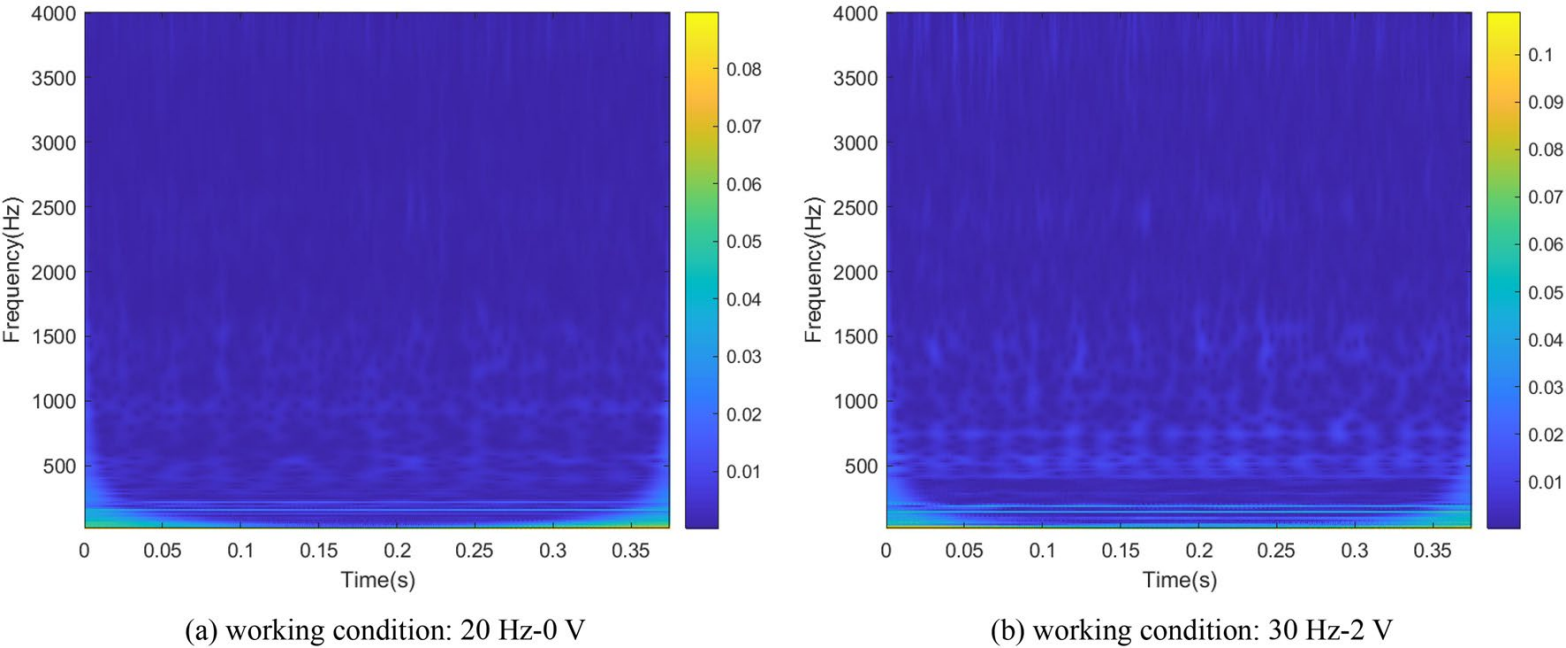
The wavelet time–frequency images of bearing signals.

**Figure 7:**
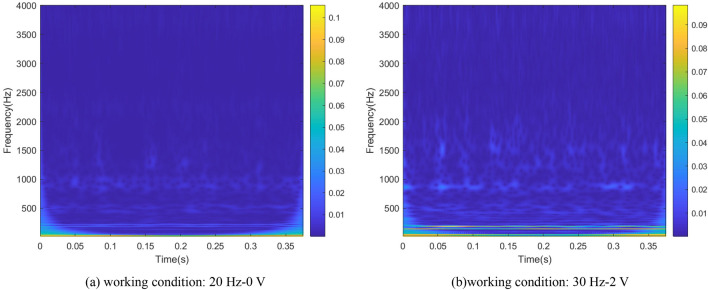
The wavelet time–frequency images of gear signals.

Wavelet transform is used to process vibration signals, mapping the original signal to 2D space. The wavelet time–frequency images of bearing and gear are obtained, as shown in Figs. 6 and 7.

### Parameter settings

The entire experiment is performed under the ubuntu 18.04 operating system, applying Python 3.8 and Pytorch 1.8 framework. It runs on a computer of an Intel (R) Xeon(R) Gold 6330 processor and a NVIDIA GeForce RTX 3090 GPU. During the experiment, Adaptive Moment Estimation (Adam) is used to update the training parameters of all models. Cross entropy is used to calculate loss values. The dropout is set to 0.2. Batch Size affects the generalization performance and convergence speed. Too small batch size could lead to a large impact on the training process. Too large could not achieve the ideal accuracy for a limited number of epochs^35^. Therefore, the Batch Size is set to 32. In the initial stage, a higher learning rate can quickly approach the optimal solution. The learning rate decay enables the model to make large weight adjustment at the initial training stage. It can perform more precise parameter adjustments near the optimal solution in the subsequent stages. The initial learning rate is set to 0.01. The learning rate decay strategy is used to optimize training process. The accuracy of the validation is set as an indicator. The learning rate is adjusted when the accuracy no longer rises. The experimental variable settings are shown in Table 3.

**Table 3:** Experimental variable definition and settings.

Variables	Definitions	Values
Batch Size	Number of samples need to be processed per batch	32
Initial learning rate	Learning rate setting at the initial training	0.01
Learning rate decay	Automatically adjust learning rate by a decrease of 0.1	0.1
Dropout	Randomly delete neurons	0.2

### Experiment and result analysis

#### Experimental verification of attention mechanism

To explore the fault diagnosis effectiveness of EfficientnetV2 network integrated with LECA, the classification accuracy of EfficientNetV2 combined with SE, ECA and LECA is compared and analyzed respectively. Bearing and gear datasets at 20 Hz-0 V working conditions are used for verification. Each model is trained for 10 times to mitigate the influence of random initial values. The average is taken. The comprehensive performance of the models is shown in Table 4.

**Table 4:** Bearing and gear fault diagnosis accuracy of models based on three attention mechanisms.

	SE-EfficientNetV2 (%)	ECA-EfficientNetV2 (%)	LECA-EfficientNetV2 (%)
Bearing	94.63	95.5	99.38
Gear	95.23	97	99.75

From Table 4, the accuracy of the three models is all above 90% on both datasets. The diagnostic performance of LECA-EfficientNetV2 is the best among all comparative methods. The model accuracy rises reasonably after considering the multi-scale input. A small size of convolution kernel could extract richer features. The accuracy reaches 99.38% and 99.75% respectively, an improvement of about 3% based on ECA module. This demonstrates the effectiveness of the LECA module.

**Figure 8:**
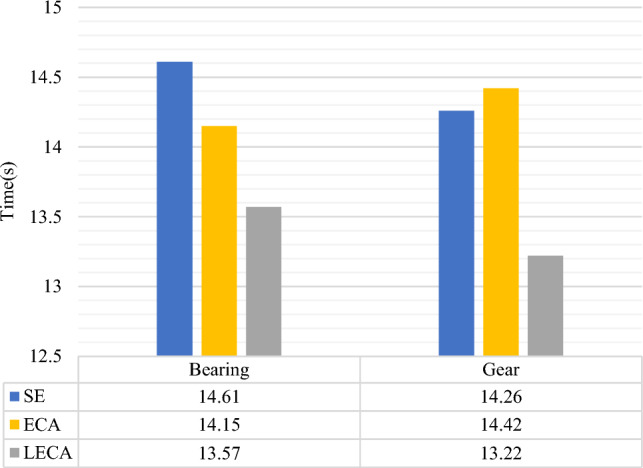
The fault diagnosis time for models based on three attention mechanisms.

In terms of fault diagnosis efficiency, SE-EfficientNetV2 has the longest diagnosis time, with an average iteration time of about 14.61 s. SE has two fully-connected layers and high computational costs. The kernel size of LECA module is smaller than ECA. The LECA-EfficientNetV2 spends the shortest diagnosis time, with about 13.57 s, as shown in Fig. 8. By comprehensive comparison, with 50 iterations, the LECA-EfficientNetV2 model has the shortest diagnosis time and the highest accuracy.

**Figure 9:**
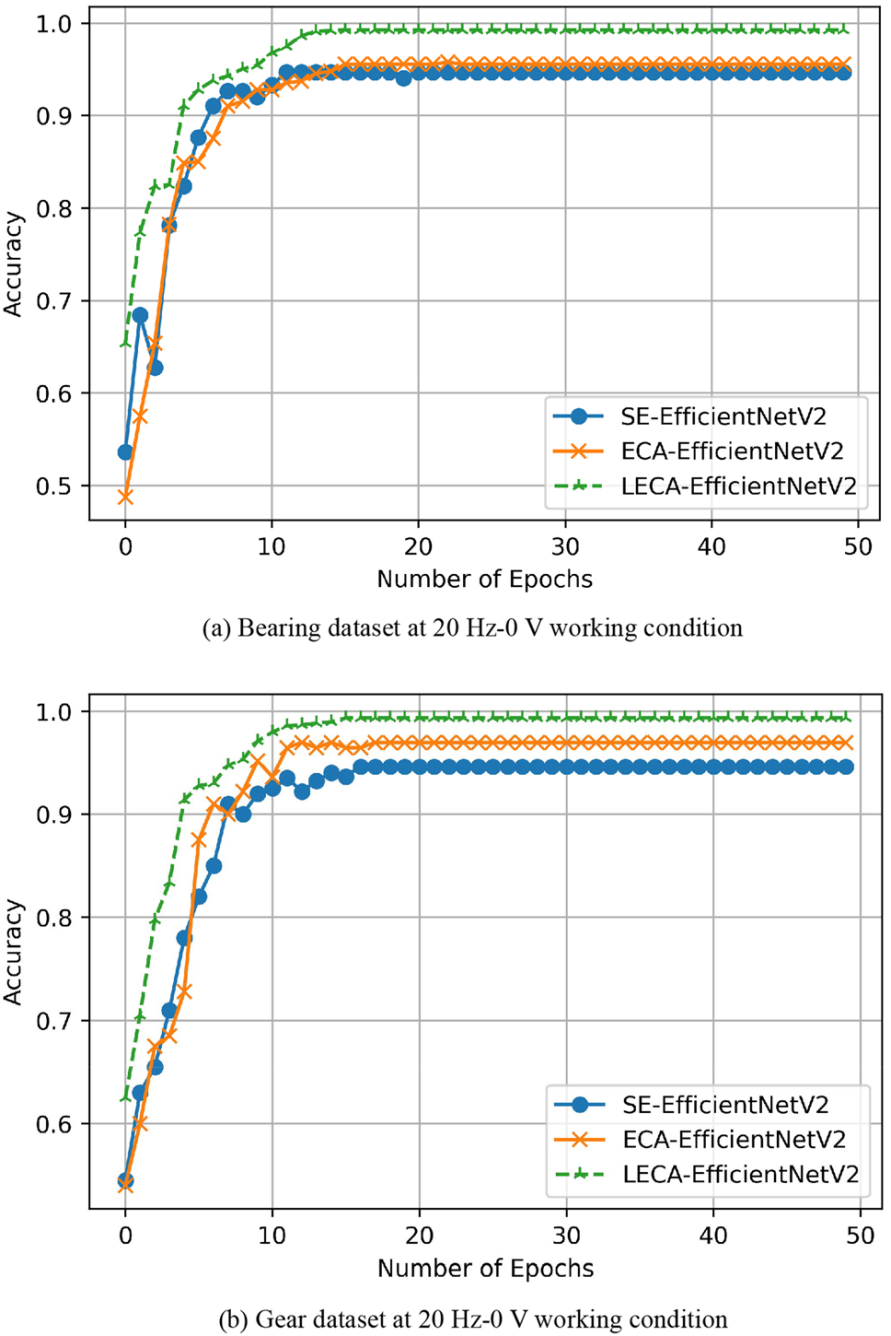
Accuracy curves of models based on three attention mechanisms for 50 iterations.

To further evaluate the superiority of the three models, Fig. 9 presents a validation accuracy curve of two experimental subjects for 50 iterations. Compared with the networks based on SE and ECA, the accuracy of LECA-EfficientNetV2 model always more than 97% after about ten iterations, which further demonstrates the model integrated with LECA module could better complete gearbox fault diagnosis.

**Figure 10:**
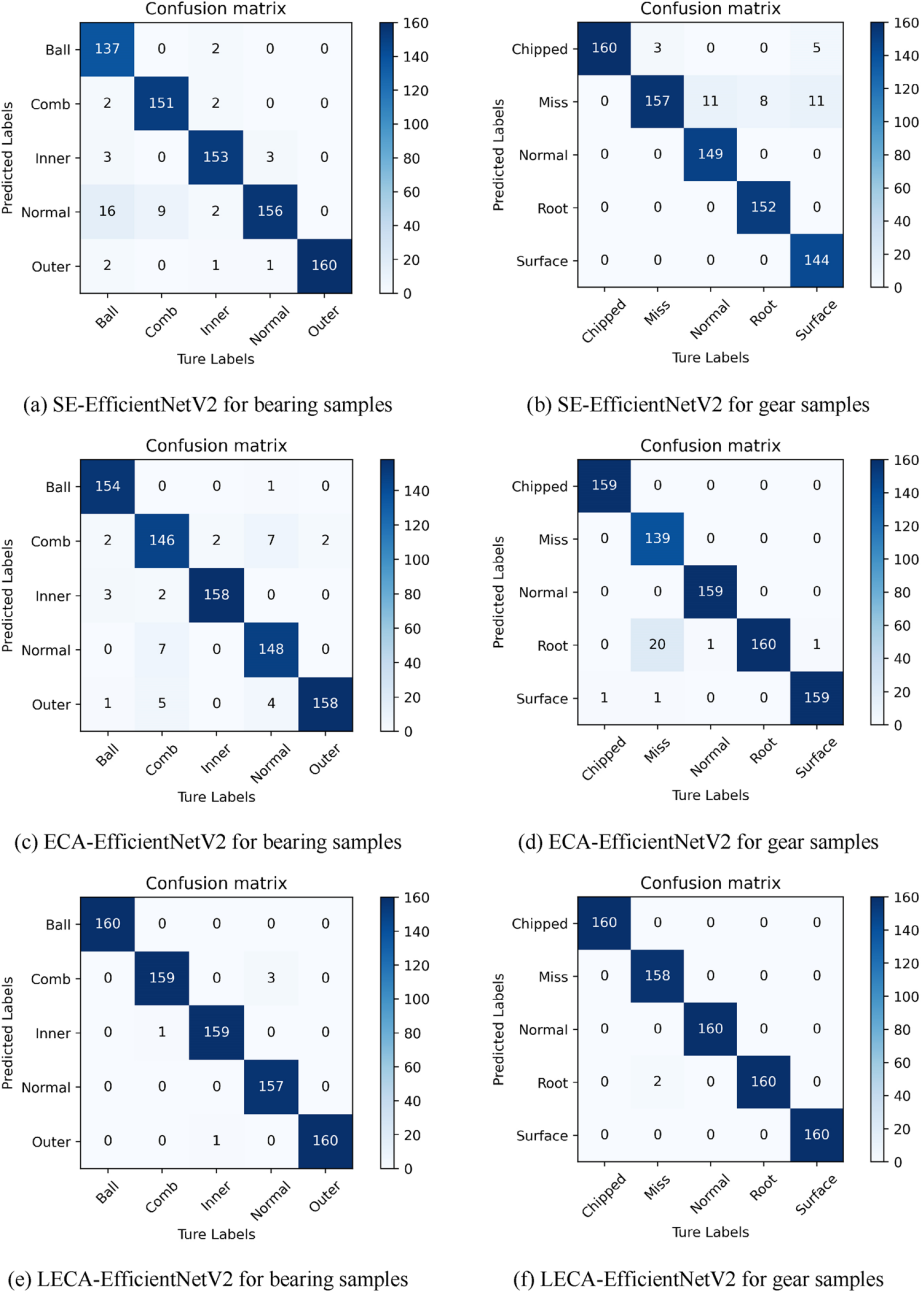
Confusion matrix for bearing and gear samples at 20 Hz-0 V working condition.

To deeply explore the reasons for false classification of samples, the paper presents the validation results of bearing and gear data under 20 Hz-0 V working condition in the form of confusion matrix, as shown in Fig. 10. The recognition capacity of SE-EfficientNetV2 on Ball and Surface faults are relatively poor. The classification effect of ECA-EfficientNetV2 on Comb and Miss faults need to be improved. In contrast, LECA-EfficientNetV2 network greatly improves the recognition accuracy on different samples. Only a few samples are misclassified. Some Normal samples are misclassified as Comb on the bearing dataset. A few Miss samples are considered as Root on gear dataset. The recognition precision reaches almost 100% on other fault types. This proves once again the excellent fault feature learning capacity of LECA-EfficientNetV2 network. Therefore, the further research is to establish a transfer learning network based on the LECA-EfficientNetV2 network.

#### Experimental verification of transfer learning fault diagnosis

The previous experiments were conducted on a dataset with sufficient fault samples. To achieve high-accuracy fault diagnosis for untrained small samples, transfer learning method is introduced and different types of transfer learning fault diagnosis tasks are set. After the network is trained on source domain dataset, the lower structures of the network are frozen and the higher structures of the network are fine-tuned with 250 target domain samples. Then 750 validation samples from target domain are used to estimate the classification capacity.

To verify the performance of the proposed network, the fault diagnosis results are compared with seven other models, namely Vgg13^36^, ResNet50^37^, MobileNetV3-L^38^, EfficientnetV1-b0^39^, EfficientnetV2-S^33^, GhostNetV2^40^, FasterNet-T2^41^ network. The above models are trained same as LECA-EfficientNetV2 network. To ensure the reliability of experimental results, the average value of 10 experiments is taken as result. The detailed transfer experiments are shown in Table 5. T1 represents the transfer of bearing fault diagnosis knowledge learned from source domain (20 Hz-0 V) to target domain (30 Hz-2 V). T3 represents the transfer of bearing diagnosis knowledge learned form source domain (20 Hz-0 V) to gear target domain (20 Hz-0 V).

**Table 5:** Transfer learning fault diagnosis experiment settings under different working conditions and components.

Task	Subject	Source domain	Target domain	Transferring method	
				LECA-EfficientNetV2	Other models
T1	Bearing	20 Hz-0 V	30 Hz-2 V	Freeze the first convolutional layer and the four Fused-MBConv modules. Fine-tune the other modules.	Freeze the top five convolution modules. Fine-tune the other modules.
T2	Gear	20 Hz-0 V	30 Hz-2 V		
T3	Bearing-Gear	20 Hz-0 V	20 Hz-0 V		
T4	Bearing-Gear	20 Hz-0 V	30 Hz-2 V		

**Figure 11:**
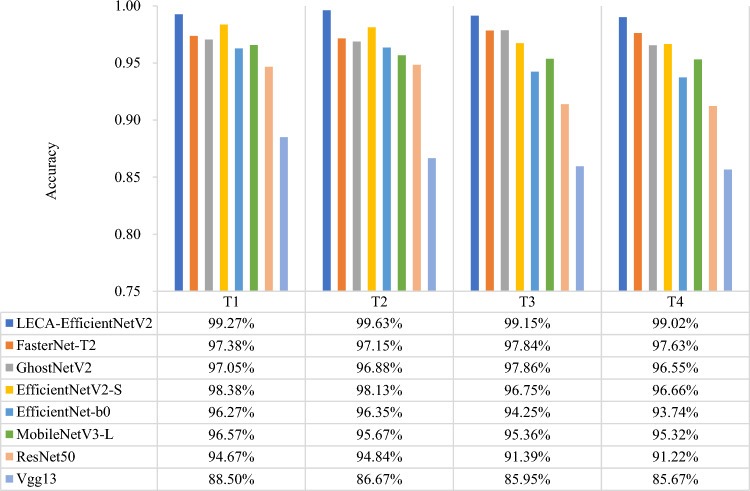
Classification accuracy of eight transfer learning fault diagnosis models in four tasks.

The classification accuracy of eight transfer learning fault diagnosis models is shown in Fig. 11. The results present the proposed models have stronger transfer feature learning ability than other models. The accuracy is 99.27% and 99.63% in T1 and T2 respectively. It proves the model is effective in diagnosing small sample faults under variable working condition. The accuracy of T3 and T4 is 99.15% and 99.02%. This method can achieve cross-component fault diagnosis and has good generalization. Among the comparative methods, The accuracy of EfficientNet series network is worse than proposed method in four tasks. It proves the model combining multi-scale feature input and LECA module can obtain richer signal information. The accuracy of FasterNet-T2 is similar to GhostNetV2, both above 97%. The Vgg13 has bad generalization ability and the worst diagnostic effect. The above results demonstrate the method can effectively extract fault features under different working conditions and components.

**Figure 12:**
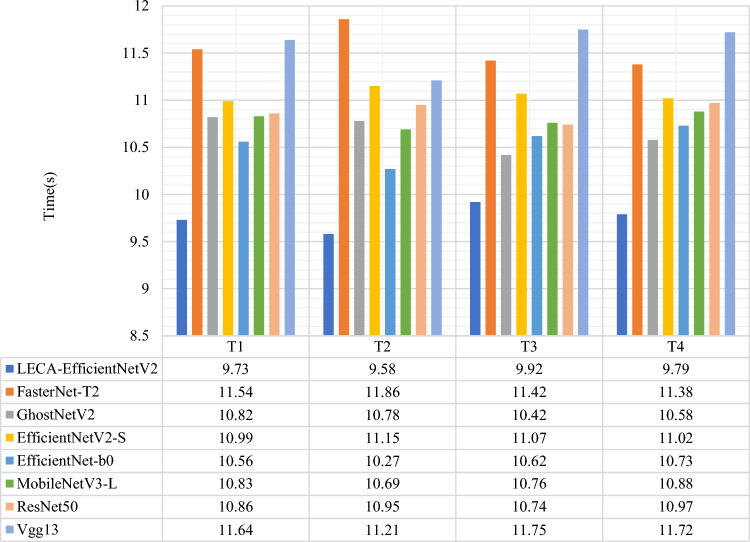
Fault diagnosis time of eight transfer learning fault diagnosis models in four tasks.

The fault diagnosis time of eight transfer learning models is shown in Fig. 12. The results show the proposed model achieves the shortest diagnosis time in four tasks. The diagnosis time is 9.73 s, 9.58 s, 9.92 s and 9.79 s in four tasks. It can realize the fast fault diagnosis in varying working condition and cross components. In addition to training time, FLoating-point Operations (FLOPs) and Parameters (Params) are usually regarded as indicators to evaluate the complexity of the model. FLOPs presents the number of floating-point operations. Params is the number of parameters of the model^35^. The complexity of eight network is shown in Table 6. The parameters of LECA-EfficientNetV2 is the smallest, but FLOPs is not the lowest. It could be related to the depth of the network, different convolution and other parameter settings. Combined with the fault diagnosis time of the model, LECA-EfficientNetV2 meets the requirements for model lightweight.

**Table 6:** The FLOPs and Params of eight models.

Model	FLOPs(G)	Params(M)
LECA-EfficientNetV2	0.98	1.19
FasterNet-T2	1.91	13.71
GhostNetV2	0.18	4.88
EfficientNetV2-S	2.88	20.18
EfficientNet-b0	0.41	4.01
MobileNetV3-L	0.37	4.21
ResNet50	4.12	23.52
Vgg13	11.38	128.98

**Figure 13:**
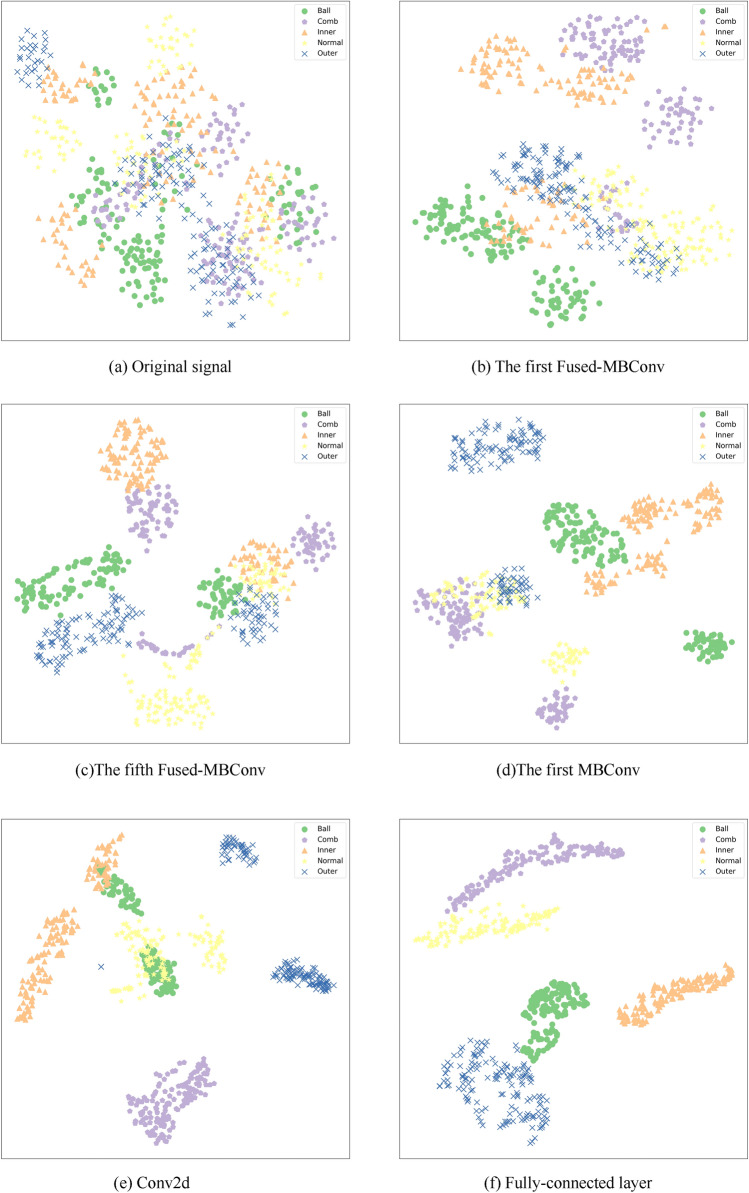
The visualization of samples feature distribution variation using t-SEN.

To observe the distribution variation process of fault data intuitively, this paper uses the t-SEN method to visualize the classification process of bearing data in T1 tasks. The detailed feature distribution is shown in Fig. 13. The dimensionality reduction visualization shows, without classification, the feature distributions of various fault signals are obviously mixed and difficult to distinguish. With the further training of the model, there are already relatively obvious five kinds of distributions in the fully-connected layer.

## Conclusion

This paper proposes a new gearbox fault diagnosis method based on lightweight channel attention mechanism and transfer learning. The method could solve the problem of bad fault diagnosis performance caused by large sample distribution difference and limited samples. The bearing and gear datasets are used to verify the classification and generalization capacity of the proposed model. The conclusions are as follows.

(1)LECA-EfficientNetV2 has been proven to get 99.38% and 99.75% accuracy on bearing and gear samples, respectively. The fault diagnosis time is 13.57 s and 13.22 s. Compared with SE-EfficientNetV2 and ECA-EfficientNetV2, LECA-EfficientNetV2 has the best diagnostic effect on both datasets. It could extract more detailed features and effectively complete gearbox fault diagnosis.

(2)The transfer learning experiments present LECA-EfficientNetV2 has the best diagnostic performance and generalization ability under different gearbox working conditions and components. The computational cost shows that proposed method could meet the requirement for model lightweight. The proposed method can realize fast and accurate classification of gearbox faults. It is of great significance to solve the problem of small samples in practical engineering applications.

Since this paper only explores two components and the validation datasets are completely balanced. However, in industrial environment, the sample imbalance problem is prominent. The aspects will be further explored in the future: (1)Further expand the application scope of LECA-EfficientNetV2 to enhance model generalization ability. (2)Further study the model performance in imbalanced datasets and maintain high accuracy.

## Data Availability

The data may be available from the corresponding author upon request.
